# Improving patient discharge and reducing hospital readmissions by using Intervention Mapping

**DOI:** 10.1186/1472-6963-14-389

**Published:** 2014-09-13

**Authors:** Gijs Hesselink, Marieke Zegers, Myrra Vernooij-Dassen, Paul Barach, Cor Kalkman, Maria Flink, Gunnar Ön, Mariann Olsson, Susanne Bergenbrant, Carola Orrego, Rosa Suñol, Giulio Toccafondi, Francesco Venneri, Ewa Dudzik-Urbaniak, Basia Kutryba, Lisette Schoonhoven, Hub Wollersheim, on behalf of the European HANDOVER Research Collaborative

**Affiliations:** Radboud University Medical Center, Scientific Institute for Quality of Healthcare (IQ healthcare), 114 IQ healthcare, P.O. Box 9101, 6500 HB Nijmegen, The Netherlands; Radboud University Medical Center, Kalorama Foundation, Nijmegen, The Netherlands; Department of Primary Care, Radboud University Medical Center, Nijmegen, The Netherlands; Patient Safety Center, University Medical Center Utrecht, Utrecht, The Netherlands; Department of Health Studies, University of Stavanger, Stavanger, Norway; University College Cork, Cork, Ireland; Department of Neurobiology, Care Sciences and Society, Karolinska Institutet, Stockholm, Sweden; Department of Social Work, Karolinska University Hospital, Stockholm, Sweden; Department of Clinical Science, Intervention and Technology, Karolinska Institutet, Stockholm, Sweden; Quality and Patient Safety, Karolinska University Hospital, Stockholm, Sweden; Department of Emergency Medicine, Karolinska University Hospital, Stockholm, Sweden; Avedis Donabedian Institute, Universidad Autónoma de Barcelona, Barcelona, Spain; Clinical Risk Management and Patient Safety Centre, Tuscany region, Italy; National Center for Quality Assessment in Health Care, Krakow, Poland

**Keywords:** Patient handoff, Patient discharge, Patient readmission, Intervention mapping, Adverse events

## Abstract

**Background:**

There is a growing impetus to reorganize the hospital discharge process to reduce avoidable readmissions and costs. The aim of this study was to provide insight into hospital discharge problems and underlying causes, and to give an overview of solutions that guide providers and policy-makers in improving hospital discharge.

**Methods:**

The Intervention Mapping framework was used. First, a problem analysis studying the scale, causes, and consequences of ineffective hospital discharge was carried out. The analysis was based on primary data from 26 focus group interviews and 321 individual interviews with patients and relatives, and involved hospital and community care providers. Second, improvements in terms of intervention outcomes, performance objectives and change objectives were specified. Third, 220 experts were consulted and a systematic review of effective discharge interventions was carried out to select theory-based methods and practical strategies required to achieve change and better performance.

**Results:**

Ineffective discharge is related to factors at the level of the individual care provider, the patient, the relationship between providers, and the organisational and technical support for care providers. Providers can reduce hospital readmission rates and adverse events by focusing on high-quality discharge information, well-coordinated care, and direct and timely communication with their counterpart colleagues. Patients, or their carers, should participate in the discharge process and be well aware of their health status and treatment. Assessment by hospital care providers whether discharge information is accurate and understood by patients and their community counterparts, are important examples of overcoming identified barriers to effective discharge. Discharge templates, medication reconciliation, a liaison nurse or pharmacist, regular site visits and teach-back are identified as effective and promising strategies to achieve the desired behavioural and environmental change.

**Conclusions:**

This study provides a comprehensive guiding framework for providers and policy-makers to improve patient handover from hospital to primary care.

**Electronic supplementary material:**

The online version of this article (doi:10.1186/1472-6963-14-389) contains supplementary material, which is available to authorized users.

## Background

Patients still experience needless harm and often struggle to have their voices heard, processes are not as efficient as they could be, and costs continue to rise at alarming rates while quality issues remain. A shorter length of hospital stay, the decrease in work-hours of health care providers, and the increasing number of patient transitions between departments and institutions requires effective patient handovers, especially those of frail patients with comorbidities [1]. Continuity of care at patient discharge from the hospital is a critical aspect of high quality patient care [2, 3]. Highly reliable care requires close cooperation between care providers across organisational boundaries, thereby establishing an interdisciplinary network [4]. Unfortunately, incomplete or incorrect information and communication errors between hospital care providers and the multiple receiving parties often increase the chance of adverse events. These may ultimately lead to life threatening situations, avoidable treatments, unplanned re-hospitalisations [5, 6], and extra costs [7–9].

Although studies have identified discharge problems in the social, organisational, linguistic and technical context [10–12], there is insufficient, evidence driven insights into more effective solutions. The effectiveness of most interventions is highly variable and limited in daily practice. Explanations for these disappointing results include the difficulty of changing providers behaviour and existing practices, non-optimal intervention strategies, inadequate resources devoted to evaluating the impact of interventions, and inadequate methods to design and evaluate interventions [13–15]. A systematic approach for translating discharge problems into customised solutions is lacking. Many clinical intervention developers select their strategies intuitively. Effective interventions need to be theory- and evidence based, and targeted at specific behavioural and environmental factors [16, 17].

The aim of our study was to systematically develop a guiding framework to more effective design of interventions that support care providers and policy-makers to improve patient handovers from the hospital to primary care.

## Methods

Intervention mapping (IM) is a systematic, iterative six-step process that helps to develop an intervention, based on theoretical, empirical and practical information [18]. The steps are summarised in Table 1. IM was originally used effectively in the health promotion domain to develop programs for smoking cessatation [19], stroke prevention [20], asthma management [21], HIV prevention [22], and leg ulcer management [23]. We modified the IM terminology in order to apply it to the quality improvement domain.

**Table 1:** Intervention mapping steps, objectives and methods*

Steps		Objectives	Methods
**1.**	**Problem analysis**	▪ Gain insight into health problem, quality of care, underlying causes and target population	▪ Problem analysis using PRECEDE-PROCEED model;
			▪ Analysis based on:
			- Literature research
			- Individual interviews (n = 321)
			- Focus group interviews (n = 26)
			- Process maps (n = 5)
			- Artifact analyses (n = 5)
			- Ishikawa (fishbone) diagrams (n = 5)
**2.**	**Identify intervention outcomes, performance objectives and change objectives**	▪ State intervention outcomes	▪ Use evidence from literature and empirical data from problem analysis (step 1)
		▪ Specify performance objectives	
		▪ Select important and changeable determinants	▪ Input from experts in the field of patient handover (healthcare providers, and organizational, social and health scientists)
		▪ Develop matrices with change objectives based on performance objectives and determinants of suboptimal hospital discharge	
**3.**	**Select theory-based methods and strategies**	▪ Identify and select theoretical methods	▪ Literature search on theory-based methods
		▪ Select evidence-based interventions and design of practical strategies	▪ Input from experts (n = 220)
		▪ Ensure that interventions and strategies address change objectives	
			▪ Systematic literature review on evidence based discharge interventions
			▪ Additional search for experience based practical strategies
			▪ Matching methods and practical strategies with determinants and performance objectives (step 1 and 2)
**4.**	**Develop an intervention**	▪ Provide suggestions for developing an intervention	▪ Input from literature search and experts
**5.**	**Implementation**	▪ Provide suggestions for writing an implementation plan	▪ Literature search of implementation strategies and tools
**6.**	**Evaluation**	▪ Provide suggestions for writing an evaluation plan	▪ Literature search on methods for effect and process evaluation on complex interventions

*Adapted from Bartholomew et al. [18].

### Step 1: Problem analysis

We structured the problem analysis by using the PRECEDE PROCEED model [24] (see Additional file 1), to analyse and describe the scale, causes, and consequences of the health problem and to identify the target population.

#### Procedure and participants

A literature search on the frequency and consequences of ineffective hospital discharge problems was performed [25]. We performed a large qualitative study on patient handovers between acute care hospitals and primary care in five countries, i.e. The Netherlands, Spain, Poland, Sweden, and Italy, to identify the behavioural and environmental determinants influencing ineffective hospital discharge [10–12]. The study adhered to the RATS (Relevance, Appropriateness, Transparency, Soundness) guidelines for qualitative studies. Data collection and analysis consisted of multi-method qualitative research including individual and focus group interviews [26], process maps, artefact analyses [10–12], and Ishikawa diagrams [27] (Table 1). The discharged patients and their care providers were recruited using general and country-specific inclusion criteria (see Additional file 2). The study was approved by the ethics committee of the University Medical Center Utrecht — Medical Ethics Committee. Patients were asked for informed consent.

### Step 2: Identify intervention outcomes, performance objectives and change objectives

In step 2, we identified the desired outcomes of the intervention and formulated specific performance objectives for the target population, such as writing a complete, accurate and timely discharge letter by the hospital physician. This resulted in a step-by-step checklist of what needs to be accomplished in order to obtain the desired outcomes [28].

It is important to identify what steps need to be tweaked in order to affect the performance objective, and ultimately the intervention outcome [28]. We identified the most important determinants (e.g., lack of knowledge and understanding between hospital and primary care providers) that need to be changed and combined these with performance objectives to formulate our change objectives. These change objectives specified who and what will change as a result of the intervention.

#### Procedure and participants

A literature search of the desired outcomes of the intervention was conducted [25]. The performance objectives and matrices of change objectives were discussed in a multidisciplinary study panel (n = 5) that included experts in health-, social- and organisational sciences. Members of the European HANDOVER Research Collaborative (n = 15 experts in the field of handover and health care providers) prioritised using a survey the large number of determinants of importance on a 5-point Likert scale.

### Step 3: Selection of theory-based methods and strategies

We selected theory-based methods that relate to the change objectives in step 2. These methods were required to change the behavioural and environmental determinants of ineffective hospital discharge. Subsequently, these methods were translated into practical strategies.

#### Procedure and participants

Theory-based methods were identified from our literature search and mainly found in overviews provided by Bartholemew et al. [18], Achterberg et al. [29], and Grol et al. [30]. A total of 220 international researchers, policy-makers and regulators in the field of quality and safety in healthcare, healthcare providers and patient representatives were consulted about their experiences with successful strategies or promising ideas during three expert meetings in 2010–2011 [31]. A systematic review of randomised controlled trials (RCTs) of the effects of discharge interventions provided an overview of evidence-based strategies [32]. The systematic review was performed in accordance with the PRISMA guidelines. An additional literature search was performed to identify promising strategies that were not included in the systematic review (e.g., evaluated with a weaker study design than RCTs) or not evaluated yet (e.g., local initiatives). The strategies were selected by the study panel after 11 iterative discussion sessions based on the findings from the systematic review, the experiences of the experts and the additional literature search.

### Step 4: Develop an intervention

In this step, we provide suggestions for the design of the intervention by considering the target group and local setting [18]. The intervention studies identified in step 3 were classified independently by two researchers (GH and MZ) according to the Oxford Centre for Evidence-Based Medicine - Levels of Evidence from 2009 onward [33].

### Steps 5 and 6: Implementation and Evaluation

We made suggestions for developing an implementation plan for accomplishing program adoption, and for evaluating the effects and feasibility of the intervention program. The suggestions were based on literature regarding effective implementation strategies [17, 30, 34–36], existing implementation toolboxes [37, 38], and a literature review on methods to evaluate complex interventions in health care [35, 39, 40].

## Results

### Step 1: Problem analysis

**Figure 1:**
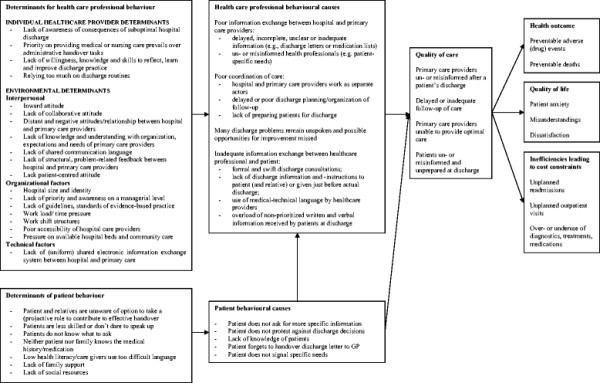
**Model of suboptimal hospital discharge: overview of the health problem, causes and their determinants.**

The health problem and the underlying causes are presented in Figure 1. The published studies demonstrate that one in five patients experience an adverse event within 3 weeks after hospital discharge, of which one in three was considered preventable [41]. Three per cent of the adverse events led to permanent disability, including death. The one month unplanned readmission rates varied between 13% [42], and 20% [43]. Unnecessary hospital readmissions lead to considerable suffering, harm and extra costs. Friedman and Basu estimated hospital costs for preventable readmissions during 6 months at about $730 million [7]. Jencks estimated total hospital costs at $44 billion per year for rehospitalisations among Medicare patients within 30 days of hospital discharge [44].

We found that ineffective handovers that lead to patient readmissions are caused by poor information exchange, poor coordination of care and poor communication between hospital and primary care providers, and between care providers and patients. The underlying causes include attitudinal and behavioural factors (e.g., lack of understanding of the needs of the counterpart, a distant relationship and a lack of collaborative attitude between hospital and primary care providers), organisational factors (e.g., lack of guidelines), technical factors (lack of a shared electronic information system) or patient factors (e.g., patients are less skilled or don’t dare to speak up). All the identified causes and their underlying factors are summarised in Figure 1.

### Step 2: Matrices of change objectives

#### Intervention outcomes and performance objectives

Measurable and feasible endpoints to evaluate the discharge process are hospital readmission rates and adverse events rates after the hospital discharge.

All performance objectives are listed in Table 2. It is important for healthcare providers to transfer high-quality discharge information to primary care providers and patients. For example, using discharge letters that are complete (i.e., no redundant/irrelevant or missed information), accurate and understandable (i.e., structured presentation of information, explanation of abbreviations jargon), and patients being informed at discharge in plain language. Regarding coordination of care, healthcare providers are expected to have organised and accurate follow-up services at patient’s discharge in a timely manner and tailored to the patient’s preferences and psychosocial needs (e.g., assessment of home setting, social risks and support). Examples of performance objectives for discharge communication are hospital care providers being accessible for primary care providers or patients and exchanging discharge information in time to support primary care providers or patients.

**Table 2:** **Performance objectives for healthcare providers and patients**

**Healthcare providers**	
Discharge information	1a. Complete discharge information
	1b. Clear discharge information
	1c. Accurate discharge information
Coordination of care	2a. Ensure that follow-up services are being organized at actual discharge
	2b. Tailor follow-up care to patient needs and preferences
	2c. Organize timely and accurate follow-up
Discharge communication	3a. Seek direct/personal contact with primary care counterpart
	3b. Discharge information easily accessible to counterpart care providers and patients (and relatives)
	3c. Exchange discharge information on time to primary care counterparts
	3d. Inform patient (and relatives) personally and in timely manner
**Patients**	
Participation in discharge process	4. Contribute, if capable, to the continuity of care in the discharge process
Awareness of health status and treatment	5. Well aware about medical history and medication use, diagnosis/indication and (side) effects of the treatment, post discharge appointments, scheduled tests and (pending) test results

Patients are, if capable, expected to contribute to the continuity of care by participating in the discharge process (e.g., by handing over a discharge letter to their GP after being discharged), and by being well aware of their health status (e.g., medical and medication history) and treatment plan.

#### Selected determinants and change objectives

The most important determinants (as perceived by experts in the field of patient handovers and described in step 2 of the methods) were classified according to the individual professional, interpersonal, organisational, technical and patient levels. Combining the performance objectives with the selected determinants resulted in two matrices with change objectives for healthcare providers and patients, which interventions need to target. The matrices are presented in Additional file 3.

### Step 3: Selecting theory-based methods and strategies

Our literature review identified a raft of change methods, such as knowledge transfer, active listening and guided practice from the Social Cognitive Theory (SCT) [45], consciousness raising from the Transtheoretical Model [46, 47], shifting perspectives and interpersonal contact from the Intergroup Contact Theory [48] and standardised working processes from the SCT and Rational Decision-making theories [30] as influencers of the behavioural and environmental determinants of ineffective hospital discharge. Goal-setting and implementation intentions were derived from theories of Goal Directed Behaviour [49, 50], and multi-disciplinary collaboration and case management from theories of Integrated Care [51]. These theory-based methods were subsequently operationalised into practical strategies and corresponding activities and materials for the targeted population [52–76] as shown in Table 3.

**Table 3:** **Overview of change determinants, theory-based methods, strategies and practical applications, and evidence**

Determinants and change objectives	Theory-based methods	Examples of strategies/ practical applications	Examples of activities and materials	References*	Evidence†
*Individual healthcare provider*					
Aware of the consequences of suboptimal hospital discharge	Knowledge transfer/Active learning	Education in the medical and nursing curriculum	Lectures on patient handover and exercises with workbook and online materials (e.g., communication skills and discharge letter requirements)	52	3a
Perceive handover administrative tasks as important part of patient discharge care and act accordingly	Stimulus control/ Reinforcement	Punishment by financial penalties; visual electronic reminders	Red, orange and green flags indicating status of discharge letter and planning; visualization of deadline for sending discharge letter	NF	NA
*Interpersonal*					
Outward focus by hospital-based care providers to ensure continuity of care after discharge	Integrated care	Post-discharge monitoring of follow-up	Standard post-discharge telephone call or home visit to the patient to evaluate follow-up, provide additional instructions and answer questions	53	1a
Hospital and primary care provider collaborative during the discharge process	Integrated care/ Intergroup contact/ Case management	Case conference	Hospital or community-based face-to-face or telephone meetings between hospital and primary care providers	54-57	1b
		Liaison person	Designated care provider coordinating hospital discharge, follow-up care and the communication between hospital and primary care providers	58-60	1b
Knowledge and understanding of the primary care organization, expectations and needs	Team building/ Intergroup contact/ Shifting perspective	Meetings between hospital and primary care providers to increase mutual understanding and respect between both parties	Focus group sessions, regular meetings and site visits to get to know each other, to learn each other’s organization and needs and to identify improvement opportunities	61	1b
Structural, problem-related feedback between hospital and primary care providers	Stimulus control	Means to facilitate and stimulate structural feedback	Standard feedback form and return envelop along with discharge letter send to primary care providers	NF	NA
Patient-centered attitude	Modeling/ Individualization	Use of plain, patient-friendly, nonmedical language	Discharge summary in language that is understandable for patients and relatives	62	1b
	Active listening	Teach back	Care provider checks if patients received all discharge information needed and if they understood the received information	63	2b
*Organizational*					
Guidelines and standards of evidence-based practice	Standardized working processes	Standardized discharge letter (e.g. templates, formats)	Templates, formats, required (web-based) fields, clinical decision-support, pick lists	64-66	1b
		Standardized discharge planning	Guidelines, protocols, checklists for discharge planning, organizing follow-up	67-68	1b
		Medication reconciliation	Standardised medication reconciliation checklist/medication discrepancy tool/ reconciliation by (liaison) pharmacist	54,57,65-67,69-71	1b
*Technical*					
Shared electronic information exchange system	Multi-disciplinarycollaboration	Shared electronic patient information system	Electronic notifications to primary care providers to inform them about patient hospital visits and to provide them (web-based) access to available discharge information	65,66,71-73	1b
*Patient and relative*					
Participation in the discharge process	Self- management/ Guided practice	Encouraging and facilitating patients in self-management skills	Provide patient with discharge record (e.g., active problem list, medication, allergies, patient concerns) owned and maintained by the patient to facilitate cross-site information transfer	62,74,75	1b
Skills and dare to speak up	Coaching/ Guided practice	Encouragement to assert a more active role during discharge	Question form for patients	74	1b
Understanding of medical history and/or medication	Guided practice/ Knowledge transfer	Medication counseling at the hospital at discharge or at the patient’s home	Visits by a pharmacist counselor	76	1b

NF = not found; NA = not available.

*The majority of the references relate to interventions or a component of a studied intervention program with an aim to improve hospital discharge. Other types of interventions (e.g., improving clinical handovers within the hospital) were also used as references in case they were considered to be relevant and appropriate for improving hospital discharge.

†Grading of evidence, adapted and adjusted from the Oxford Centre for Evidence-based Medicine Levels of Evidence^33^: 1b = systematic review or meta-analysis of randomized controlled trials (RCTs); 1a = RCT of good-moderate quality or sufficient size and consistency; 3-4 = comparative trials (non-randomized, cohort studies, patient-control studies); 4 = non-comparative studies; 5 = Expert committee reports, opinions and/or clinical experience of respected authorities.

### Step 4: Develop an intervention

We formulated a wide variety of change objectives at the individual clinician and patient levels, the interpersonal level, organisational and technical levels that need to be considered in order to tackle ineffective handovers at discharge more reliably (Additional file 3). Given these change objectives the intervention likely needs to be multi-faceted and needs to be tailored to the needs encountered in the local setting. Table 3 shows a framework with examples of strategies and related materials and activities guiding healthcare providers and policy makers in the development of their intervention. The list of all identified strategies and related materials, level of evidence and references are available upon request.

Many interventions were evaluated in well-designed studies. For example, the use of standardised discharge practices such as the use of discharge letter templates, discharge planning guidelines and medication reconciliation checklists are effective strategies [65–67, 70]. The use of a shared electronic patient information platform facilitates discharge communication between hospital and primary care providers [66, 71–73]. There is evidence demonstrating that the patient’s role in the discharge process is enhanced by the provision of written and verbal discharge information and by assistance and guidance in self-management (e.g., discharge counselling, follow-up calls or home-based visits and a patient discharge record or question form) [74, 76]. However, many promising interventions have not been evaluated properly or were tested using weak study designs. For example, the effects of lectures and exercises on discharge practice in the medical curriculum, and regular group discussions involving hospital and primary care providers are largely unknown [52].

Moreover, there is limited evidence on the effects of reinforcement by using discharge planning reminders, mandatory administrative tasks or financial incentives and penalties [77].

Insight also lacks into the effects of strategies to increase care provider reflections on discharge practices (e.g., use of a standardised feedback form, video reflection, role play or simulation of discharge consultations) [52] and regarding the use of teach-back to check the patient’s understanding of their medical and medication history [63, 78].

### Steps 5 and 6: Implementation and evaluation

Commitment from and ownership by the target group is essential to successful implementation [79, 80]. The awareness among end users is enhanced when they are directly involved in the development or modification of the innovation, in mounting the implementation plan, and in selecting the implementation strategies to be used [35].

Moreover, uptake of policies and protocols, reimbursement and the consideration of patients’ preferences are necessary for a sustainable implementation [81].

Strategies that address the barriers to change are required to implement interventions in daily practice [36]. Most theories on implementing interventions in health care emphasise that an analysis of the barriers to change practice is a prerequisite to selecting or developing an effective implementation strategy [17]. An implementation plan should be developed specifically after selecting the implementation strategies to tackle the barriers. This plan should be compatible with the target group and settings in which the implementation will take place. Good management and planning of implementation activities (i.e., what, when, where, how and by whom) also appears to be a requisite for successful implementation of innovations in patient care [35].

Formative and summative effect evaluation should be carried out using hospital readmission and adverse events as defined patient outcome effects to evaluate whether the intervention led to the desired degree of change. The formulated performance objectives in step 2 can be operationalised in measurable process indicators, for example by assessing the proportion of patients discharged with a complete discharge letter and assessing the proportion of patients discharged after medication reconciliation.

A process evaluation should be performed to understand the effect, success or failure of the intervention and to get an impression of its feasibility, generalizability and its acceptability in the target population. The process evaluation gives insight into the black box of the implementation process and can explain the variation in results in evaluating interventions. The activities carried out as part of the intervention, the actual exposure of participants to these activities, and their experience of these activities should be studied [40].

## Discussion

Effective hospital discharge and reducing patient readmission rates are influenced by the behaviours of care providers and patients and their environmental context. Our findings demonstrate the existence of a large number of determinants for (in)effective discharge that underscore the complexity of the discharge process. Therefore, improving hospital discharge requires a multi-component, multi-level intervention (“bundle”) instead of trying to find a “magic bullet” single intervention.

An extensive overview of theory-based methods and practical strategies suitable for improving patient handover skills and healthcare provider and patient behaviour in the discharge process was systematically created based on the scale, causes, and consequences of ineffective hospital discharge presented in our study. Most interventions were aimed at improving the organisational and technical aspects of the discharge process. There is a lack of evidence-based interventions on improving healthcare provider skills by means of handover training and evidence-based guidance. Moreover, effective interventions for changing the individual healthcare provider’s and patient’s competencies, awareness and attitudes (e.g., via education, reminders or teach-back), and the relationship between providers (e.g., via frequent informal meeting between hospital and primary care providers and reflexive feedback) are lacking. All this despite our overwhelming data demonstrating that awareness, attitudes and skills are key factors for improving hospital discharge. We found a gap between the discharge improvement needs and the evidence-based interventions that are suitable to address these needs. The lack of evidence about the effectiveness of interventions may be attributed to the difficulty of measuring attitudes and their effects on healthcare performance [82–84].

This study is supported by earlier research and discharge programs in the United States: i.e., the RED (“ReEngineerd Discharge”) project [69, 85], the Care Transitions Program [86] and BOOST (Better Outcomes for Older adults through Safe Transitions) [87]. An important strength of our study is the deliberate assessment of determinants and interventions that affect the discharge process. Qualitative input provides comprehensive insights into a variety of determinants. Our empirical data, results of a systematic literature review, theories of social behaviour and multiple consultation rounds of a broad group of 324 experts (researchers, policy-makers, inspectors) in the field of quality and safety in healthcare, healthcare providers and patient representatives [31], provided useful input for the selection of change methods, practical strategies and related evidence.

A limitation of the study is our focus on the micro-level excluding other key factors for change. The possible barriers and facilitators at a macro- and meso-levels, i.e., financial and legal obligations or constrains were not included. Moreover, the relationships between the identified determinants and theoretical-based methods and strategies were hypothetical.

However, the determinants were systematically and theory-driven and linked to practical strategies using the IM method and were not intuitively chosen.

## Conclusions

This study provides a comprehensive overview of patient discharge problems and underlying causes. It provides a guiding framework including theory-based strategies and practical tools to support care providers and policy-makers in their efforts to select and implement interventions on a more rational basis. Intervention mapping is a powerful method for care providers and policy makers to assess and prioritise intervention strategies and tailor them to the needs of individual facilities and healthcare systems. The next step for care providers and policy-makers is to look carefully into the discharge problems in their own local settings and to select appropriate solutions for improving hospital discharge effectively.

## Electronic supplementary material

Additional file 1: **Modified model based on PRECEDE-PROCEED concept and the theory of planned behavior** (DOCX 33 KB)

Additional file 2: **Study Population Inclusion and Exclusion Criteria.** (DOCX 15 KB)

Additional file 3: **Matrix of change objectives.** (DOCX 20 KB)
