# Supplementary material for: Improving patient discharge and reducing hospital readmissions by using Intervention Mapping
Source: BMC Health Serv Res. 2014 Sep 13;14:389. doi: 10.1186/1472-6963-14-389 (PMC4175223; doi:10.1186/1472-6963-14-389)
Supplement: Supplementary file 1 — Additional file 1: Modified model based on PRECEDE-PROCEED concept and the theory of planned behavior (DOCX 33 KB) [file 12913_2014_3488_MOESM1_ESM.docx]

Societal

Community

Organisational

Interpersonal

External factors

Professional factors

Skills

Intention

Attitudes

Experienced social influences*

Self-efficacy†

**Professional behaviour**

Quality of care

Health

Quality of life

* Subjectively perceived social influences (as opposed to interpersonal external factors, which are objective social influences).

† Trust in own capacities to perform according to standards of good care.
