# Supplementary material for: Improving patient discharge and reducing hospital readmissions by using Intervention Mapping
Source: BMC Health Serv Res. 2014 Sep 13;14:389. doi: 10.1186/1472-6963-14-389 (PMC4175223; doi:10.1186/1472-6963-14-389)
Supplement: Supplementary file 2 — Additional file 2: Study Population Inclusion and Exclusion Criteria. (DOCX 15 KB) [file 12913_2014_3488_MOESM2_ESM.docx]

| **Additional file 2. Study Population Inclusion and Exclusion Criteria** | |
| --- | --- |
| **Inclusion** | |
| Patients | 18 years old+ |
|  | Admitted to internal medicine, pulmonary, cardiology or (vascular) surgical wards |
|  | Any of the following diagnoses: diabetes mellitus, asthma, COPD, chronic heart failure |
|  | Prescribed 6+ drugs |
|  | Recruited consecutively at the point of their hospital discharge |
|  | Discharged to the community (i.e. home or nursing home) |
| Hospital physicians and nurses | Internal medicine, pulmonary diseases, cardiology or (vascular) surgical wards |
| GPs and community nurses | Representing the communities to which the patients were discharged |
| **Exclusion** | |
| Patients referred to other care units within the hospital prior to their discharge home or discharge to another country. | |
| GP=general practitioner; COPD=chronic obstructive pulmonary disease. | |
