# Supplementary material for: Improving patient discharge and reducing hospital readmissions by using Intervention Mapping
Source: BMC Health Serv Res. 2014 Sep 13;14:389. doi: 10.1186/1472-6963-14-389 (PMC4175223; doi:10.1186/1472-6963-14-389)
Supplement: Supplementary file 3 — Additional file 3: Matrix of change objectives. (DOCX 20 KB) [file 12913_2014_3488_MOESM3_ESM.docx]

| **Additional file 3. Matrix of change objectives** | | | | | | | | | | | | | |
| --- | --- | --- | --- | --- | --- | --- | --- | --- | --- | --- | --- | --- | --- |
| **CARE PROVIDER BEHAVIOR, ORGANISATIONAL AND TECHNICAL CONTEXT** | | | | | | | | | | | | | |
| **DTs** | **Individual care provider** | | | **Interpersonal** | | | | | | **Organisational** | | | **Technical** |
| **POs** | *Unawareness of consequences of ineffective hospital discharge* | | *Priority of care prevails over administrative handover tasks* | *Hospital-based care providers’ inward focus* | *Lack of a collaborative attitude* | *Lack of knowledge/*  *understanding of PC expectations and needs* | *Lack of structural, problem-related feedback* | *Patient-centred attitude* | | *Lack of EB guidelines/*  *standards* | *Work shift structure of hospital personnel* | *Poor accessibility of hospital care providers* | *Lack of shared IT system* |
| Discharge information | Explain and discuss the potential consequences of a incomplete, unclear, inaccurate DL  Recognise the need for a clearly written DL and ML | | Appraise writing DLs as essential part of providing care  Plan sufficient time to write DLs | Recognise the importance of sharing information and to make it easy available for PC providers after discharge | Provide verbal explanation of discharge information to PC counterpart when needed | Recognise if information is sufficient and understandable for the PC counterpart  Identify what information is needed to handover care | Provide and value feedback when discharge information is not handed over complete, in time, clear and/or accurate | Check if patients received all information they needed/preferred  Recall if patients understood the received information  Identify ways to inform patients about discharge and follow-up in an understandable way | | Standardise discharge information structure and content  Check if exchanged information is correct (no duplications/ variations) | Recognise who/when is first responsible for a patient’s discharge  Describe what discharge information is already written down and what still needs to be done |  |  |
| Coordination of care | Explain and discuss the potential consequences of improper assessment of follow-up needs | | Recognise the need to take time to assess follow-up needs of patients  Plan sufficient time to organise follow-up services  Recognise the need to plan discharge and organise follow-up services in time | Monitor discharged patients in the community | Arrange a meeting with PC counterpart to discuss patient follow-up needs  Allocate tasks and responsibilities clearly between hospital and PC  PC providers review information of hospital counterparts seriously | Recognise and value the PC organisation, needs and capabilities  Recognise available follow-up services (e.g. support, medication) | Provide and value feedback when follow-up is not clear enough or does not meet the needs and preferences of PC providers and patients | Inspect patient specific follow-up needs and wishes  Arrange shared-decision making of follow-up needs between care providers and with patient/relative | | Standardise DP and organisation of follow-up services | Organise a constant group of care providers around the patient nearing discharge  Assign one person responsible for coordinating discharge  Describe follow-up that is organised and what needs to be done |  | Share patient information of all involved health care providers in one database |
| Discharge communication | Explain and discuss the potential consequences of a delayed DL | | Recognising the moment when a DL really needs to be sent away in order to continue care in the community  Plan sufficient time to write DLs | Recognise the importance of being accessible to counterpart  Alert PC providers on nearing patient discharge | Recognise that personal/direct contact between a hospital and PC provider about a patient’s discharge can be beneficial for an adequate and timely follow-up of care | Inform counterpart what time is most appropriate to be called  Define situations when hospital care provider needs to take the initiative to call the PC counterpart and vice versa | Provide and value structural feedback when counterpart is hard to reach | Make care providers better reachable for discharged patients  Recognise the need to inform the patient/relative personally and on time  Schedule time for informing patient about discharge and follow-up | | Standardise communication between different healthcare providers  Check if counterpart understood the information (recall) | Assign one person who coordinates discharge communication between hospital and PC providers | Improve the organisational means to make care providers better reachable for the counterpart during or after patient hospitalisation | Provide access to patient information for hospital and PC providers at any time |
| **PATIENT AND RELATIVE** | | | | | | | | | | | | | |
| **DTs**  **POs** | | **Unawareness of important role in the discharge process** | | | | **Lack of skills and dare to speak up** | | | **Lack of understanding of medical history and/or medication** | | | | |
| Participation in discharge process | | Recognise that they can contribute to an effective hospital discharge  Handing over the DL to responsible PC provider  Carrying an up–to-date medication list | | | | Signal specific needs and preferences at hospital discharge | | | Identify possible side effects and indications that health condition is worsening and know how to respond | | | | |
| Awareness of health status and treatment | | Recognise the need to learn about medical history, medication use, diagnosis and (side) effects of treatment | | | | Ask questions concerning hospitalisation, discharge and follow-up care  Ask for more explanation/clarification if felt needed | | | Health care providers inform patients and check if patients understood the received information | | | | |
| DL=discharge letter; DP=discharge planning; DT=determinants; ML=medication list; PC=primary care; PO=performance objectives. | | | | | | | | | | | | | |
